# Impact of Inherited Thrombophilia in Women with Obstetric Antiphospholipid Syndrome: A Single-Center Study and Literature Review

**DOI:** 10.3390/biomedicines12061174

**Published:** 2024-05-25

**Authors:** Blanca Camacho Sáez, Víctor M. Martínez-Taboada, Ana Merino, Alejandra Comins-Boo, Belén González-Mesones, Sara Del Barrio-Longarela, Leyre Riancho-Zarrabeitia, Marcos López-Hoyos, José L. Hernández

**Affiliations:** 1 Division of Rheumatology, Hospital Marqués de Valdecilla-IDIVAL, 39008 Santander, Spain; blanca.camacho@alumnos.unican.es (B.C.S.); vmartinezt64@gmail.com (V.M.M.-T.); 2 Departamento de Medicina y Psiquiatría, Universidad de Cantabria, 39011 Santander, Spain; hernandezjluis@gmail.com; 3 Division of Obstetrics and Gynecology, Hospital Marqués de Valdecilla, 39008 Santander, Spain; anaisabelmerino@gmail.com (A.M.); saradelbarrio@hotmail.com (S.D.B.-L.); 4 Immunology Department, Hospital Universitario Marqués de Valdecilla-IDIVAL, 39008 Santander, Spain; alejandra.comins@scsalud.es; 5 Heamatology Department, Hospital Universitario Marqués de Valdecilla-IDIVAL, 39008 Santander, Spain; belen.gonzalezmesones@scsalud.es; 6 Rheumatology Department, Hospital Sierrallana-IDIVAL, 39300 Torrelavega, Spain; leyre.riancho@scsalud.es; 7 Departamento de Biología Molecular, Universidad de Cantabria, 39011 Santander, Spain; 8 Department of Internal Medicine, Hospital Marqués de Valdecilla-IDIVAL, 39008 Santander, Spain

**Keywords:** inherited thrombophilia, pregnancy, obstetric morbidity, fetal loss, antiphospholipid syndrome, antiphospholipid antibodies

## Abstract

Inherited thrombophilia (IT) has been implicated as a potential causal factor of adverse pregnancy outcomes (APOs), including recurrent miscarriage with and without the presence of antiphospholipid syndrome (APS). The aim of this study was to assess the prevalence and impact of IT on fetal–maternal outcomes and thrombotic risk in women within the spectrum of obstetric APS. Three hundred and twenty-eight women with APS-related obstetric morbidity ever pregnant were included. Of these, 74 met the APS classification criteria, 169 were non-criteria (NC)-APS, and 85 were seronegative (SN)-APS. Patients with other autoimmune diseases were excluded. APOs included early pregnancy loss, fetal death, preeclampsia, abruptio placentae, and preterm birth. Successful pregnancy was defined as the achievement of a live newborn. A literature search was also performed. The mean age of the overall group was 33.9 ± 5.3 years, and the patients were followed up for 35 (11–79) months. During the study period, there were 1332 pregnancies. Nearly 14% of the patients had an associated IT. IT patients more frequently received the standard-of-care (SoC) therapy. The presence of IT was not associated with worse maternal–fetal outcomes in patients treated with SoC treatment. Overall, IT patients had a lower frequency of newborns without treatment, especially those without definite APS. In addition, IT did not increase the risk of thrombosis during pregnancy or the postpartum period. A detailed analysis of the literature review identified only four publications related to our study and did not show conclusive evidence of the impact of IT on patients with obstetric APS. The group of women with APS-related obstetric morbidity and IT who did not receive treatment, especially those without definite APS, had a worse prognosis in terms of a live birth. However, with SoC therapy, the prognosis is similar in those patients without IT. The association of IT with APS does not seem to predispose to the development of thrombosis during pregnancy and/or the postpartum period.

## 1. Introduction

Antiphospholipid syndrome (APS) is an autoimmune disease characterized by thrombotic and/or obstetric events associated with the presence of antiphospholipid antibodies (aPLs) [1]. Diagnosing APS requires both clinical and serological criteria. However, patients who do not strictly meet the classification criteria may present with what have been called “clinical manifestations related to APS” or with an inconclusive serological profile not included in the criteria definition. This is especially relevant in the subgroup of patients with obstetric APS [2]. 

Although there is a clear association between obstetric complications and the presence of aPLs, women at reproductive age may present other related comorbidities that may complicate their pregnancy wishes [3]. More specifically, in addition to classic cardiovascular risk factors, such as obesity, smoking, or hypertension, other conditions, such as thyroid diseases, local uterine disorders, or inherited thrombophilia (IT), have been related to adverse events during pregnancy [4,5,6,7]. Thrombophilia has been implicated through different mechanisms, such as microvasculature thrombosis and inhibition of extravillous trophoblast differentiation, as a potential causal factor of adverse pregnancy outcomes (APOs), including recurrent miscarriage [8]. Low-dose aspirin (LDA) and low-molecular-weight heparin (LMWH) are recommended by the main therapeutic guidelines to prevent APOs in patients with APS [9,10,11,12]. Nevertheless, the therapeutic scheme for patients with isolated IT remains controversial, and a recent clinical trial in women with recurrent miscarriages and IT has not shown the benefit of LMWH [13]. In this regard, there is scarce information on the prevalence and direct impact on maternal–fetal prognosis in patients with concomitant aPLs and IT. In the largest study to date, no significant differences were found either in terms of adverse events during pregnancy or in the development of thrombotic events in IT carriers, compared to women who only had aPLs [14]. This study included patients with APS and non-criteria (NC)-APS from a multicenter European registry [14]. In fact, their conclusions were confirmed in a later study, where a decrease in the prevalence of IT was observed [15]. As expected, most of the included patients received combined treatment with LDA and LMWH, achieving a favorable obstetric outcome in a significant proportion of pregnancies.

Taking into account all these considerations and the paucity of data published to date, our study aimed to assess the prevalence and impact of IT on fetal–maternal outcomes and thrombotic risk in women within the spectrum of obstetric APS.

## 2. Materials and Methods

### 2.1. Study Participants

**Figure 1:**
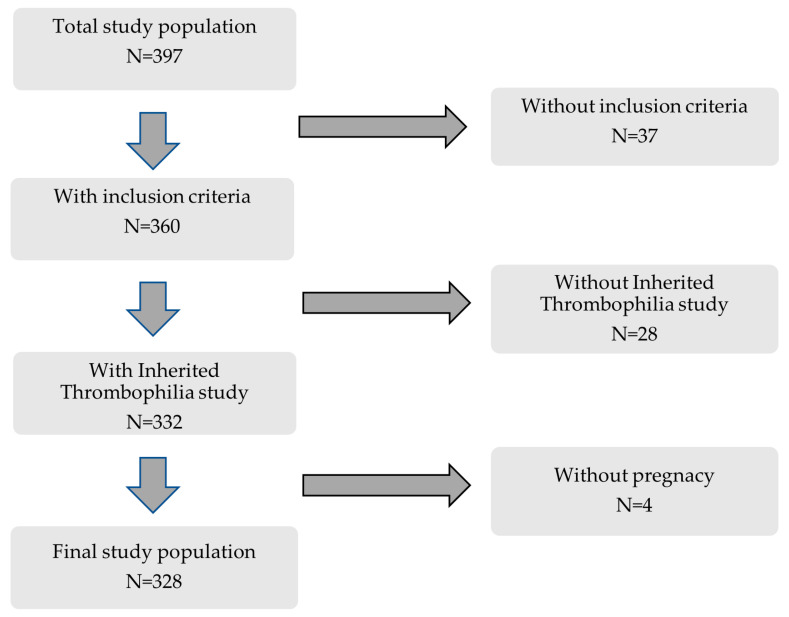
Flowchart of patients included in this study.

This retrospective cohort study included 397 consecutive women followed at the Autoimmune Diseases Pregnancy Clinic, a multidisciplinary unit of a teaching tertiary care hospital, between 2005 and 2022. Inclusion criteria were (a) patients included in previously well-defined clinical–serological subgroups [16], (b) IT study available, and (c) at least one clinical pregnancy (Figure 1). 

As shown in Table 1, 328 patients were categorized into the following groups: (a) Criteria APS (*n* = 74): patients were classified according to the Sidney classification criteria [1]. (b) NC-APS (*n* = 169): patients who did not meet strict clinical and serological classification criteria for the disease. According to Alijotas-Reigh et al. [2], these patients were divided into the following subgroups: Subgroup A (*n* = 34): non-criteria obstetric morbidity related to APS and inconclusive serology; Subgroup B (*n* = 63): clinical manifestations included in the criteria and inconclusive serology; and Subgroup C (*n* = 72): non-criteria obstetric morbidity related to APS and serology included into the classification criteria. (c) SN-APS (*n* = 85): clinical manifestations included in the criteria and persistently negative serology. The main clinical and serological characteristics of the study groups are shown in Supplementary Table S1.

Women who fulfilled the classification criteria for rheumatic autoimmune diseases other than APS were excluded. The information collected from individual cases was completely anonymized, and the study was approved by the Ethics Committee of Cantabria (internal code: 2023.033).

### 2.2. Data Collection

Data were collected using a prespecified standardized questionnaire and stored in a computerized database. We assessed the following clinical variables:

- Demographic and general characteristics: age, sex, body mass index (BMI), current/past tobacco use, high blood pressure (equal or greater than 140/90 mm Hg or being on antihypertensive agents), dyslipidemia (serum total cholesterol or triglyceride levels greater than 230 mg/dl and 150 mg/dl respectively or being on lipid-lowering drugs), diabetes mellitus (according to the ADA criteria) past or present family (<50 years), or personal history of thrombotic disease.
- Comorbidities: three main entities associated with pregnancy outcomes were also recorded: (a) inherited thrombophilia (see Section 2.4); (b) thyroid disease (history of hypo/hyperthyroidism or the presence of confirmed specific autoantibodies); and (c) obstetric comorbidity (local uterine abnormalities, endometriosis, and polycystic ovary syndrome).

### 2.3. Autoantibody Assessment

The presence of the following antibodies and aPL isotypes was quantified by commercial enzyme immunoassay in solid phase (ELISA; Orgentec Diagnostika GmbH, Mainz, Germany): anti-cardiolipin antibodies (aCLs) and anti-beta2 glycoprotein I antibodies (AB2GPIs) of the IgG and IgM isotypes. The results are reported as quantitative and semiquantitative values. Thus, aCLs are quantified in GPL (aCL IgG) or MPL (aCL IgM) according to the standard curve built in each test with 5 dilution points of the Harris/Sapporo standards. AB2GPI are quantified as U/mL. Only medium–high titers of aPLs were considered positive. The criteria recommended by the International Society of Thrombosis and Hemostasis Scientific (ISTH) and the Standardization Committee for the standardization of lupus anticoagulant/antiphospholipid antibodies (LAs/aPLs) were applied for the characterization of LA [17,18,19]. Inconclusive serology was defined as persistent low-titer aCLs or AB2GPIs and/or intermittent ALs, aCLs, or AB2GPIs. The serologic diagnosis was made out of the pregnancy period.

### 2.4. Inherited Thrombophilia Study

Protein C, protein S, antithrombin, MTHFR, FVQ506 (FV Leiden), and prothrombin 20210A mutation (GPM) were assessed. Natural anticoagulants were analyzed before pregnancy or at least 12 weeks post-partum. The studies were performed according to the supplier’s protocols. Genomic DNA was extracted from peripheral leukocytes from EDTA-anti-coagulated blood using a commercially available DNA isolation kit. Genotype for the factor V Leiden gene mutation (R506Q mutation) and Factor II G20210A (G20210A 20210G>A 3`UTR) mutation was determined by RT-PCR (LightMix-Roche Diagnostics/Anyplex™ II Thrombosis SNP Panel Assay-Seegene). MTHFR mutation was analyzed using real-time PCR (Genvinset MTHFR C677T-Blackhills Diagnostics Resources). Heterozygosity for MTHFR was not considered in the final analysis. Specific Assays for determining Protein C Activity (chromogenic method, Berichrom Protein C Substrate-Siemens/HemosI™ Protein C-Werfen), antithrombin (INNOVANCE® Antithrombin Assay-Siemens Healthineers/Chromogenic, HemosI™ Antithrombin-Werfen) and Free Protein S ELISA (DG-EIA PS Free-Diagnostic Grifols/latex-based Turbidimetry method, Hemosil^®^Free Protein S-Werfen), were analyzed in a Fully Automated Blood Coagulation Analyzer (CA-650; Sysmex Corporation, Kobe, Japan/TOP 550, Bedford, MA, USA, Werfen).

### 2.5. Pregnancy Morbidity Definitions

- Obstetric manifestations: (a) Sidney criteria [1] and (b) non-criteria obstetric morbidity related to APS: 1–2 early pregnancy losses (<10 weeks), preterm birth (between 34 and 36 + 6 weeks), late preeclampsia (>34 weeks), abruptio placentae, and unexplained in vitro fertilization failures (IFFs) (>2) [20].
- Pregnancy loss: early pregnancy loss (<10 weeks) and/or fetal death (>10 weeks).
- Adverse pregnancy outcomes (APOs): early pregnancy loss, fetal death, preeclampsia, abruptio placentae, and preterm birth (<37 weeks).
- Successful pregnancy was defined as the achievement of a live newborn.

### 2.6. Literature Search and Study Selection

We searched MEDLINE up to the end of May 2023, using a comprehensive search strategy that combined MeSH terms and free text for “antiphospholipid syndrome”, “antiphospholipid antibodies”, “pregnancy morbidity”, “non-criteria antiphospholipid antibodies”, “non-criteria obstetric morbidity”, “recurrent miscarriages”, “fetal death”, “premature births”, “pregnancy outcomes”, and “inherited thrombophilia”. Reference lists of all relevant studies, reviews, and letters were also searched to identify additional studies. The searches were limited to studies in humans and the English language. The result of the search strategy is shown in Supplementary Figure S1.

### 2.7. Statistical Analyses

Results were expressed as numbers (percentage), mean ± standard deviation (SD), or median and interquartile range (IQR), as appropriate. Student’s *t*-test, Mann–Whitney U-test, or one-way ANOVA was used to compare quantitative variables, and chi-squared or Fisher’s test was used to compare categorical data. A two-tailed *p*-value < 0.05 was considered statistically significant in all the calculations. IBM SPSS 28.0 was used for the statistical analyses (Armonk, NY, USA: IBM Corp).

## 3. Results

### 3.1. General Features of the Study Cohort

During the study period, 328 consecutive patients fulfilled the inclusion criteria (Figure 1 and Table 1) and had a total of 1332 pregnancies. The main characteristics of the study cohort are shown in Table 2. The mean age of the overall group was 33.9 ± 5.3 years, and the median follow-up was 35 (11–79) months. Overall, as previously described [16], the prevalence of classic cardiovascular risk factors ranged from 45% to 62% and were especially prevalent in patients with APS. In addition, the most frequent comorbidities with a potential impact on the obstetric outcome, such as IT, thyroid disease, or obstetric comorbidities, were also frequent in all study groups. After diagnosis, most women received standard-of-care (SoC) treatment with LDA and/or LMWH during subsequent pregnancies (Table 3) [9,10,11,12]. As expected, patients with IT, especially those with criteria APS received more intensive treatment than those in the other study groups.

### 3.2. Prevalence and Types of Inherited Thrombophilia

Approximately 14% of the patients had an associated IT (Table 2). As shown in Table 4, the most frequently found genetic variant was protein S deficiency (6.1%), followed by homozygous MTHFR mutation (3.7%) and MGP (2.4%). The mutations that occurred to a lesser extent were protein C deficiency (0.6%) and the FVL mutation (0.9%). On the other hand, the incidence of combined coagulopathy was only observed in 1.5% of the patients. No patient presented with antithrombin deficiency. No statistically significant differences were observed between the different study groups. Furthermore, no significant differences were observed in the serological profile in the two groups of patients with aPLs.

### 3.3. Patients with Inherited Thrombophilia without Treatment Have Worse Pregnancy Outcomes

**Figure 2:**
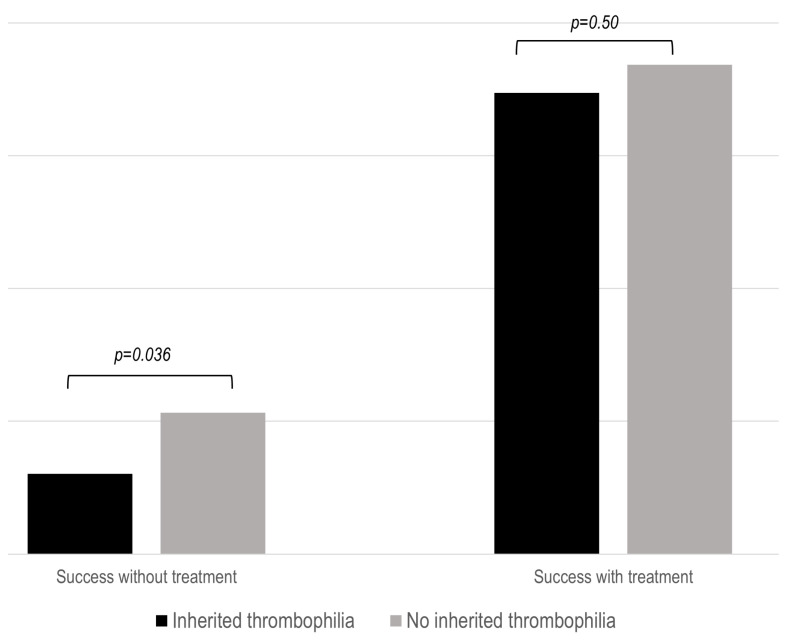
Proportion of successful pregnancy in the IT groups after standard treatment. The results show the percentage of live births compared to the number of patients with or without standard (SoC) treatment.

As previously stated, successful pregnancy was defined as the achievement of a live newborn. Untreated patients with IT had a significantly lower success rate (*p* = 0.036) during pregnancies (Figure 2). This fact was especially true in patients with NC-APS and SN-APS (Supplementary Figure S2). However, SoC therapy was associated with similar obstetric outcomes in the three study groups.

### 3.4. The increase in APOs in Patients without IT was Mainly Related to the Presence of Cardiovascular Risk Factors

The vast majority of patients had at least one APO, as expected. Those women without IT had a higher frequency of preterm birth (*p* = 0.037) and preeclampsia (*p* = 0.035) (Supplementary Table S2). In the same way that occurred with the outcome of live birth, the difference was only significant in patients who did not receive SoC treatment (Table 5). To clarify this finding, the main demographic characteristics, cardiovascular risk factors, and comorbidities were analyzed in patients with those two APOs. No significant differences were found regarding age or main comorbidities. However, cardiovascular risk factors were related to the development of APOs (Table 6). Patients with preterm births tended to have more cardiovascular risk factors (*p* = 0.08) and dyslipidemia (*p* = 0.17). Likewise, patients who developed preeclampsia also tended to have more cardiovascular risk factors (*p* = 0.08), diabetes mellitus (*p* = 0.09), and a significantly higher rate of hypertension (*p* = 0.011).

### 3.5. The Presence of IT Is not Associated with an Increase in Thrombotic Events

The patients who developed a thrombotic episode during pregnancy or the immediate postpartum period are shown in Table 7. Only four out of the 328 patients in our cohort (1.2%) (one with criteria APS, and the remaining three cases in group B) suffered a thrombotic event diagnosed by appropriate radiologic techniques (CT-scan, MRI, and Doppler ultrasound. None of these patients had an associated IT. Fifty percent of these thromboses were of arterial origin. On the other hand, 75% of these patients were under 35 years old; only one had cardiovascular risk factors at the time of thrombosis, and two pregnancies were due to in vitro fertilization techniques. Furthermore, 50% of the patients did not receive any treatment at the moment of the thrombotic episode, and the remaining were on LWMH, one at prophylactic and the other at therapeutic doses.

### 3.6. What Do We Learn from a Detailed Literature Review?

The main studies identified in the literature search are shown in Table 8. The first study to investigate the prevalence of the FVL, GPM, MTFHR, and PAI-1 mutations in patients with APS was published in 2001 by Forastiero et al. [21]. In this case–control study, the authors analyzed 105 patients, all positive for LA and/or aCL (classified as APS (*n* = 69) and NC-APS (*n* = 36)), and 200 unrelated healthy controls. The frequencies of FVL, MTHFR-677TT, and the PAI-1 4G/4G genotype were not significantly more frequent either between the aPL carrier and control groups or between the APS and NC-APS groups. However, GPM was significantly more frequent in APS patients than in controls (OR = 4.67, *p* = 0.02). In addition, this genetic variant was more prevalent in patients with APS (8.7%) than in those classified in the NC-APS group (2.8%), although the difference did not reach statistical significance. Furthermore, the prevalence of combined IT (combination of GPM or FVL with PAI-1) was significantly more frequent in patients with APS than in controls (5.8% vs. 0.5%; *p* = 0.016). This difference was not found between NC-APS patients and healthy subjects. There was also a positive, albeit not significant, trend to a higher proportion of multiple genetic defects in patients with APS compared with those without.

In 2013, Berman et al. [22] published another case–control study in which the primary objective was to determine the prevalence and clinical significance of FVL and GPM polymorphisms in patients with APS. One hundred patients with APS and a history of thrombosis were studied (77 with APS and 23 APS associated with SLE) and compared with two control groups, one that included 200 healthy individuals and another with 100 individuals with deep vein thrombosis (DVT) of the lower extremities. The FVL polymorphism was found in 1% of the cases (specifically in 1.3% of the patients with primary APS, while none of those with APS-SLE had the mutation), in 3% of healthy individuals (*p* = 0.49) and 16% of patients with first DVT (*p* < 0.0005). No significant differences were found between the different groups regarding GPM. Although patients with GPM had a higher prevalence of venous thrombosis compared to those without IT, it did not reach statistical significance (80% vs. 47.9%, *p* = 0.35).

A third study (EUROAPS project) was published in 2016 by Alijotas-Reig et al. [14]. They included women with obstetric APS, with the main objective of analyzing the prevalence and effects of IT on maternal–fetal outcomes and obstetric complications. IT data on 208 women (147 had APS and 61 NC-APS) were collected and analyzed. Sixty (24%) of them had IT, and 12/60 (20%) women with IT had more than one thrombophilia disorder. Although the prevalence of IT was relatively high, thrombotic events were rare, and no statistically significant differences were observed when comparing the groups with and without IT. Moreover, the presence of more than one IT together with aPLs did not increase the risk of adverse outcomes during pregnancy. Concerning treatment rates, no significant differences were observed between women with and without IT, revealing very good maternal and fetal outcomes when LDA plus LMWH were administered, regardless of the presence of IT. Finally, an extension of the EUROAPS project, including 1000 women with obstetric APS and published in 2019 by the same investigators [15], showed a slightly lower prevalence of IT (15.9%). No association between these entities and the rate of thrombosis was found.

## 4. Discussion

After an exhaustive literature review (Table 8), it can be concluded that, in aPL carriers, there is no clear relationship between the presence of IT and either the development of APOs or thrombotic events during pregnancy. However, the evidence is scarce and controversial, and therefore, more studies are needed to clarify this issue. Thus, in this study, we analyze the impact of IT on the obstetric outcome and thrombotic risk in a large cohort of patients belonging to the obstetric APS spectrum, including NC-APS and SN-APS. As previously reported [16], and notwithstanding the differences inherent to the definition of the study groups, the obstetric prognosis of patients on SoC therapy is very similar and overall satisfactory. However, despite appropriate treatment, a significant proportion of patients develop at least one APO.

We have observed that around 14% of the women studied had some form of IT. Supplementary Table S3 shows a comparison of the prevalence of these thrombophilias in the general population and this study [7,23,24,25,26]. A higher prevalence of protein C and S deficiency was observed in our population, whilst that of GPM, MTFHR, and antithrombin deficiency was similar to that found in the general population. Additionally, we found a lower FVL prevalence compared to the general population. These differences may be due to several factors, such as the selection of the sample, the demographic characteristics of the women studied, as well as environmental and genetic factors that may vary across populations. Furthermore, as it is well known, during pregnancy, there is a physiological deficit of protein S and an acquired resistance to activated protein C that may be related to excessive estrogen stimulation [27], and although functional determinations have been attempted outside of the period of hormonal influence, this might not always have been the case.

An interesting finding of our study is that women who did not receive treatment and presented an associated IT had a poorer prognosis concerning the most important outcome, which is a live newborn. In this regard, again, there are conflicting results in the literature on the impact of IT on obstetric outcomes [28,29,30,31,32,33,34,35]. Noteworthy, when given adequate treatment, the prognosis was similar to that of patients without IT, and this was especially true in the group of patients with IT and those in whom the symptoms and/or the serological profile are not very conclusive. However, it remains to be determined which treatment is the most appropriate. The type of treatment, either LDA alone or dual therapy with LDA plus LMWH, should be established in well-designed clinical trials. It is important to note that these differences in outcomes are not observed in patients with definite APS, probably because these women receive more intensive therapy than the other study groups or due to the impact of the aPL profile itself on the obstetric prognosis.

Another intriguing finding from our study was that patients without IT had a higher rate of third-trimester complications, specifically more preterm deliveries and PE. We found that the differences were only maintained when analyzing APOs without treatment. A detailed analysis of these patients showed that those with PE without treatment tended to have a higher prevalence of overall cardiovascular risk factors, especially more high blood pressure, and patients with preterm delivery tended to have a higher proportion of overall cardiovascular risk factors. Therefore, these results suggest that cardiovascular risk factors seem to have a predominant role in placentation disorders, whilst coagulopathy probably has little relevance. For this reason, it is essential to carefully monitor these patients during pregnancy, planning to control all cardiovascular risk factors that may harm maternal and fetal health. 

On the other hand, as has been pointed out, pregnancy entails a procoagulant state per se. However, the frequency of thromboembolic events during pregnancy, even in patients with APS, is quite low [16]. The global frequency of antenatal DVT is 0.615/1000 pregnancies in women under 35 years and almost double (1.216/1000) in those older than that. The postpartum DVT rate ranges from 0.5% to 2% depending on the population analyzed, so in women without treatment, there would be between 5 and 20 episodes of thrombosis for every 1000 pregnancies [36,37]. Nevertheless, most women receive LMWH in the postpartum period, avoiding most of these episodes. Thus, considering that 1332 pregnancies have occurred in our cohort, there will have been 3.0 thrombotic episodes for every 1000 pregnancies, 1.5 episodes per 1000 pregnancies in the antenatal period, and in the same proportion in the postpartum period. Therefore, the proportion of thrombosis in our cohort was very similar to that of the general population [36,37]. Based on our findings and some previous reports [14,15,22], it can be suggested that presenting an IT does not predispose to the development of thrombosis during pregnancy or the immediate postpartum period. It should be considered that the vast majority of patients included in our study were on SoC treatment after diagnosis (Table 3).

This study has some limitations. First of all, those inherent to a retrospective design. Additionally, it was carried out in a single center and a multidisciplinary unit specifically devoted to the treatment of obstetric complications in patients with autoimmune diseases. This means that the results cannot be extrapolated to other populations and probably to the care of pregnant patients outside specialized units. Finally, other aPLs not included in the classification criteria were not analyzed, which could have helped to better categorize the different groups, especially SN-APS and NC-APS.

We consider that this study has several advantages over previous ones. Firstly, these studies have included patients with aPLs related to other autoimmune diseases, mainly systemic lupus erythematosus [14,15,22], whereas we have excluded those patients. Thus, we could analyze a more homogeneous population of patients belonging to the clinical spectrum of APS. Secondly, the present cohort represents the whole spectrum of patients with a clinical suspicion of APS. It ranges from SN-APS to patients with primary APS, defined according to the classification criteria [1]. Moreover, we have also included patients with aPLs who present obstetric manifestations not included in these criteria that represent a very relevant subgroup in real-world clinical practice. Another strength is that we have assessed other comorbidities that could influence the overall obstetric prognosis in addition to the cardiovascular risk factors and the serological profile [5,6,7]. 

In summary, the presence of IT in patients within the clinical spectrum of APS does not seem to be associated with a worse obstetric prognosis or a greater risk of thrombosis during pregnancy or the immediate postpartum period. While treatment with LDA and/or LMWH seems effective and allows the same prognosis in treated women, those patients with IT without SoC treatment have a lower live birth rate. However, the design of this study does not allow us to determine which would be the most appropriate therapeutic option for these patients. While, in this population, SoC therapy confers a good obstetric outcome in terms of a live newborn, the frequency of APOs remains increased in a significant proportion of cases. In particular, preeclampsia presents a clear relationship with a higher prevalence of cardiovascular risk factors. It is reasonable to infer that the preconception correction or modification of these factors would contribute to a substantial improvement in the obstetric prognosis in women within the spectrum of obstetric APS.

## Figures and Tables

**Table 1:** Study groups according to the clinical and serological manifestations of the Sydney criteria and the presence of obstetric morbidity related to antiphospholipid syndrome.

Serology	Clinical Manifestations		
	Sydney Criteria	Related Obstetric Morbidity	No Manifestations
Sydney Criteria	Criteria APS *n* = 74	Subgroup C *n* = 72	
Inconclusive	Subgroup B *n* = 63	Subgroup A *n* = 34	
Negative	Seronegative APS *n* = 85		

APS: antiphospholipid syndrome.

**Table 2:** Demographic characteristics, cardiovascular risk factors, and main comorbidities in the different study groups.

	Total *n* = 328	Criteria APS *n* = 74	Non Criteria APS *n* = 169	Seronegative APS *n* = 85
Age, years ± SD	33.9 ± 5.3	33.4 ± 5.4	34.1 ± 5.7	33.9 ± 4.6
Time to diagnosis (days), median [IQR]	731 [375–1519]	964 [427–1542]	685 [279–1402] ^§^	839 [504–1807] ^§^
Follow-up, (months), median [IQR]	35 [11–79]	65 [25–158] ^# ¶^	33.5 [11–67] ^§^	23 [6–52] ^§ #^
Cardiovascular risk factors, *n* (%)	166 (50.6)	46 (62.2) ^# ¶^	82 (48.5) ^¶^	38 (44.7) ^#^
- Obesity	53 (16.2)	18 (24.3) ^# ¶^	25 (14.8) ^¶^	10 (11.8) ^#^
- Smoking	108 (32.9)	32 (43.2) ^#^	55 (32.5)	21 (24.7) ^#^
- High blood pressure	23 (7.0)	6 (8.1)	12 (7.1)	5 (5.9)
- Diabetes	9 (2.7)	1 (1.4)	3 (1.8)	5 (5.9)
- Dyslipidemia	20 (6.1)	5 (6.8)	12 (7.1)	3 (3.5)
Comorbidities, *n* (%)	114 (34.8)	20 (27.0)	63 (37.3)	31 (36.5)
- Inherited thrombophilia	45 (13.7)	9 (12.2)	21 (12.4)	15 (17.6)
- Thyroid disease	41 (12.5)	6 (8.1)	22 (13.0)	13 (15.3)
- Obstetric comorbidity	51 (15.5)	7 (9.5) ^¶^	33 (19.5) ^¶^	11 (12.9)

APS: antiphospholipid syndrome. ^§^ SN-APS vs. NC-APS: *p* < 0.05. ^#^ Criteria APS vs. SN-APS: *p* < 0.05; ^¶^ Criteria APS vs. NC-APS: *p* < 0.05.

**Table 3:** Main treatments in patients with and without inherited thrombophilia (IT).

	Total *n* = 328	IT *n* = 45	No IT *n* = 283	*p*
Standard treatment, *n* (%)	293 (89.3)	44 (97.8)	249 (88.0)	0.048
- LDA monotherapy	109 (33.2)	7 (15.6)	102 (36.0)	0.007
- LMWH	185 (56.4)	37 (82.2)	148 (52.3)	<0.001
- LDA + LMWH	175 (53.4)	33 (73.3)	142 (50.2)	0.004
Additional treatments, *n* (%)				
- Corticosteroids	45 (13.8)	9 (20.0)	36 (12.8)	0.19
- Antimalarials	17 (5.2)	2 (4.4)	15 (5.3)	0.99
- IVIGs	5 (1.6)	2 (4.4)	3 (1.1)	0.13

LDA: low-dose aspirin; LMWH: low-molecular-weight heparin; IVIGs: intravenous immunoglobulins.

**Table 4:** Inherited thrombophilia in the different study groups.

	Total *n* = 328	Criteria APS *n* = 74	Non-Criteria APS *n* = 169	SN-APS *n* = 85
Protein S deficiency, *n* (%)	20 (6.1)	6 (8.1)	8 (4.7)	6 (7.1)
Protein C deficiency, *n* (%)	2 (0.6)	0 (0.0)	2 (1.2)	0 (0.0)
Factor V Leiden, *n* (%)	3 (0.9)	2 (2.7)	1 (0.6)	0 (0.0)
MTHFR homozygous, *n* (%)	12 (3.7)	0 (0.0)	8 (4.7)	4 (4.7)
PT G20210A, *n* (%)	8 (2.4)	1 (1.4)	2 (1.2)	5 (5.9)
Combined IT, *n* (%)	5 (1.5)	1 (1.4)	3 (1.8)	1 (1.2)
Without IT, *n* (%)	283 (86.3)	65 (87.8)	148 (87.6)	70 (82.4)

IT: inherited thrombophilia; APS: antiphospholipid syndrome; MTHFR: methylenetetrahydrofolate reductase.

**Table 5:** Adverse pregnancy outcomes (APOs) in patients with and without inherited thrombophilia (IT) according to standard-of-care treatment.

	APOs without Treatment			APOs with Treatment		
	Total *n* = 328	IT *n* = 45	No IT *n* = 283	Total *n* = 328	IT *n* = 45	No IT *n* = 283
APO total, *n* (%)	283 (87.2)	35 (77.8) ^¶^	248 (87.6) ^¶^	135 (41.2)	19 (42.2)	116 (41.0)
Abortion < 10 weeks	245 (74.7)	31 (68.9)	214 (75.6)	99 (30.2)	17 (37.8)	82 (29.0)
Fetal death > 10 weeks	49 (14.9)	7 (15.6)	42 (14.8)	17 (5.2)	1 (2.2)	16 (5.7)
Preterm < 37 weeks	28 (8.5)	0 (0.0) ^§^	28 (9.9) ^§^	25 (7.6)	2 (4.4)	23 (8.2)
Abruptio placentae	4 (1.2)	0 (0.0)	4 (1.4)	3 (0.9)	1 (2.2)	2 (0.7)
Preeclampsia	25 (7.6)	0 (0.0) ^#^	25 (8.9) ^#^	16 (4.9)	1 (2.2)	15 (5.3)

IT: inherited thrombophilia; APO: adverse pregnancy outcome. ^¶^ *p* = 0.07; ^§^ *p* = 0.020; ^#^ *p* = 0.033.

**Table 6:** Cardiovascular (CV) risk factors in patients without inherited thrombophilia (IT) who developed adverse pregnancy outcomes (APOs).

	Without IT	Preterm < 37 Weeks		Preeclampsia	
	Total *n* = 283	Yes *n* = 46	No *n* = 237	Yes *n* = 37	No *n* = 246
CV risk factors, *n* (%)	146 (51.6)	29 (63.0) ^§^	117 (49.4) ^§^	24 (64.9) ^#^	122 (49.6) ^#^
- Obesity	46 (16.2)	10 (21.7)	36 (15.2)	8 (21.6)	38 (15.4)
- Smoking	96 (33.9)	17 (37.0)	79 (33.3)	13 (35.1)	83 (33.7)
- High blood pressure	24 (7.1)	4 (8.7)	17 (7.2)	7 (18.9) ^¶^	14 (5.7) ^¶^
- Diabetes	8 (2.4)	2 (4.3)	7 (3.0)	3 (8.1) ^$^	6 (2.4) ^$^
- Dyslipidemia	20 (6.0)	5 (10.9)	12 (5.1)	3 (8.1)	14 (5.7)

^§^ *p* = 0.08; ^#^ *p* = 0.08; ^¶^ *p* = 0.011; ^$^ *p* = 0.09.

**Table 7:** Main characteristics of the patients who developed a thrombotic episode during pregnancy or the immediate postpartum period.

Age, yrs	APS Group	Serology	IT	CVRF s	Time	Thrombosis	Treatment	Pregnancy
23	Criteria APS	aCLs + AB2GPI	No	Smoking	Postpartum	Arterial (stroke)	No treatment	Spontaneous
38	Group A	LA + aCLs + AB2GPI not confirmed	No	No	Postpartum	Venous (ovarian vein thrombosis)	LWMH 40 mg	IFF
34	Group B	aCLs + not confirmed	No	No	Pregnancy	Venous (lower limb thrombosis)	No treatment	IFF
27	Group B	LA + AB2GPI not confirmed	No	No	Pregnancy	Arterial (stroke)	LWMH 80 mg	Spontaneous

IT: inherited thrombophilia; APS: antiphospholipid syndrome; CVRF s: cardiovascular risk factors; IFF: in vitro fertilization technique; LWMH: low-weight-molecular heparin; APS: antiphospholipid syndrome; Group B: obstetric criteria and NC-aPL; LA: lupus anticoagulant; AB2GPI: anti-beta 2 glycoprotein I; aCLs: anti-cardiolipin antibodies.

**Table 8:** Main studies on the impact of inherited thrombophilia in patients with obstetric antiphospholipid syndrome.

Authors [Ref.]	Year	Design	Study Population	Group (*n*)	Objectives	Main Results
Forastiero R. et al. [21]	2001	Retrospective	Patients positive for LA and/or aCL classified as APS (69) and not APS (36)	*n* = 305 105 cases 200 controls	To determine the prevalence of four IT polymorphisms in patients with aPLs	The prevalence of GPM was significantly more frequent in patients with APS than in controls (*p* = 0.02). Combinations of GPM or FVL with PAI-1 were significantly more common in APS patients than in controls (5.8% vs. 0.5%, *p* = 0.016).
Berman H. et al. [22]	2013	Retrospective	Women with APS and a history of thrombosis	*n* = 400 Cases: 100 SAF SAF: 77 SAF + SLE: 23 Controls: 200 healthy 100 with 1st episode of DVT	To determine the prevalence and clinical significance of IT polymorphisms: FVL and GPP in patients with APS	The FVL variant was found in 1% of APS patients, in 3% of healthy controls (*p* = 0.49), and in 16% of patients with first DVT (*p* < 0.0005). GPM was found in 6% of APS patients, 2.5% of the healthy controls (*p* = 0.21), and 13% of the patients with DVT (*p* = 0.14).
Alijotas-Reig J. et al. [14]	2016	Retrospective Prospective	Women with obstetric APS or OM diagnosed between 2010 and 2016	*n* = 208 APS: 147 OM: 61	To analyze the prevalence and effects of IT on maternal–fetal outcomes and obstetric complications in women with aPLs	A total of 24% of the cases had an associated IT, and only 20% presented more than one thrombophilic disorder. Thrombotic events were rare, and no statistically significant differences were observed when comparing the groups with and without IT. No differences in the maternal–fetal prognosis were found.
Alijotas-Reig J. et al. [15]	2019	Retrospective Prospective	Women with obstetric APS	*n* = 1000	To analyze the clinical characteristics, laboratory data, and maternal–fetal outcomes of women with obstetric APS	A total of 15.9% of the cases had an IT. No association between IT and a high rate of thrombosis was found.

APS: antiphospholipid syndrome; aPLs: antiphospholipid antibodies; LA: lupus anticoagulant; AB2GPI: anti-beta 2 glycoprotein I; aCLs: anti-cardiolipin antibodies; IT: inherited thrombophilia; GPM: PT G20210A mutation; FVL: factor V Leiden; DVT: deep vein thrombosis; OM: obstetric morbidity.

## Data Availability

Due to research still being conducted on the project in our research group, full data are not available. Additional data are available upon reasonable request to the corresponding author.

## Supplementary Materials

The following supporting information can be downloaded at https://www.mdpi.com/article/10.3390/biomedicines12061174/s1. Figure S1. Flowchart of literature search results. Figure S2. Proportion of successful pregnancy in the study groups after standard treatment according to the different study groups. The results show the percentage of live births compared to the number of patients with or without standard (SoC) treatment. Supplementary Table S1. Clinical and serological characteristics in the different study groups. Supplementary Table S2. Adverse pregnancy outcomes (APO) in patients with and without inherited thrombophilia (IT). Supplementary Table S3. Prevalence of inherited thrombophilia (IT) in the general population [7,19,20,21,22] and in the group of the present study.
