# Supplementary material for: Impact of Inherited Thrombophilia in Women with Obstetric Antiphospholipid Syndrome: A Single-Center Study and Literature Review
Source: Biomedicines. 2024 May 25;12(6):1174. doi: 10.3390/biomedicines12061174 (PMC11201097; doi:10.3390/biomedicines12061174)
Supplement: Supplementary file 1 [file biomedicines-12-01174-s001.zip › biomedicines-2973824-supplementary.pdf]

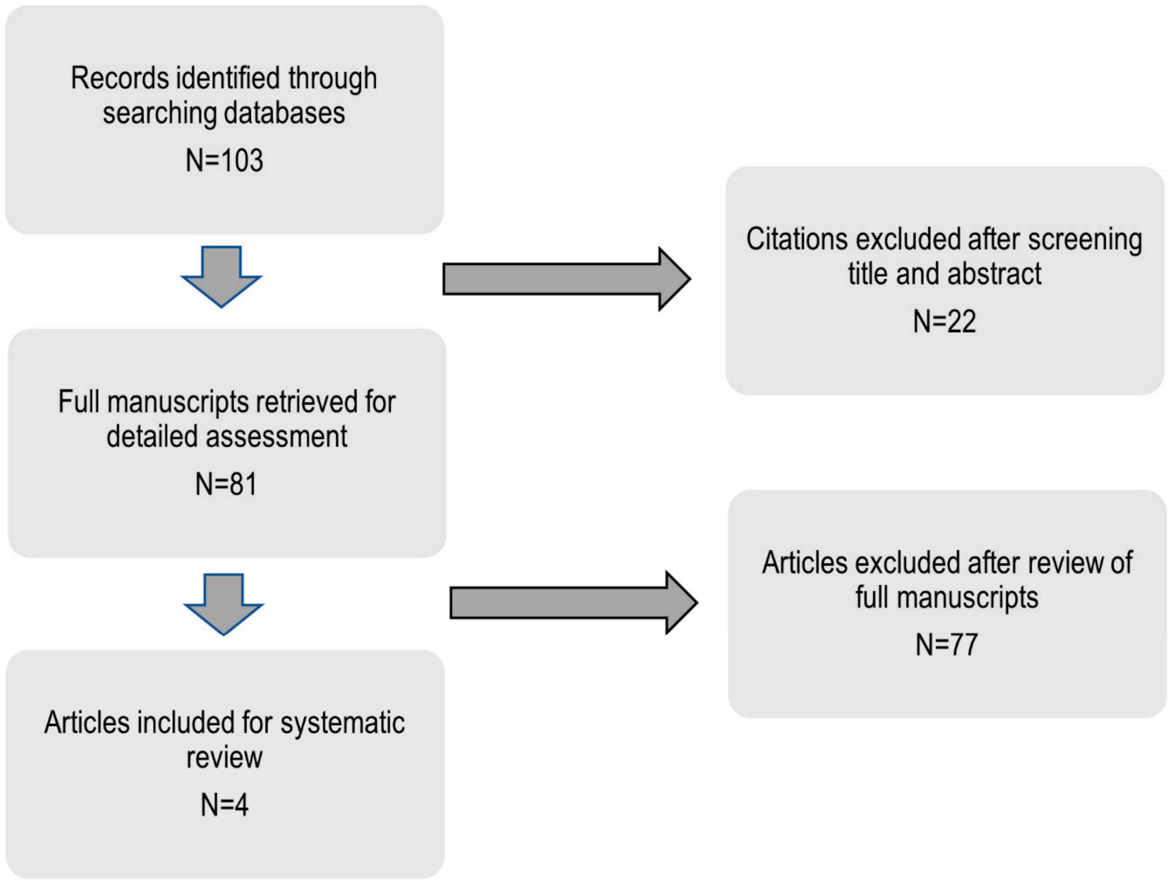

Figure S1. Flowchart of literature search results.

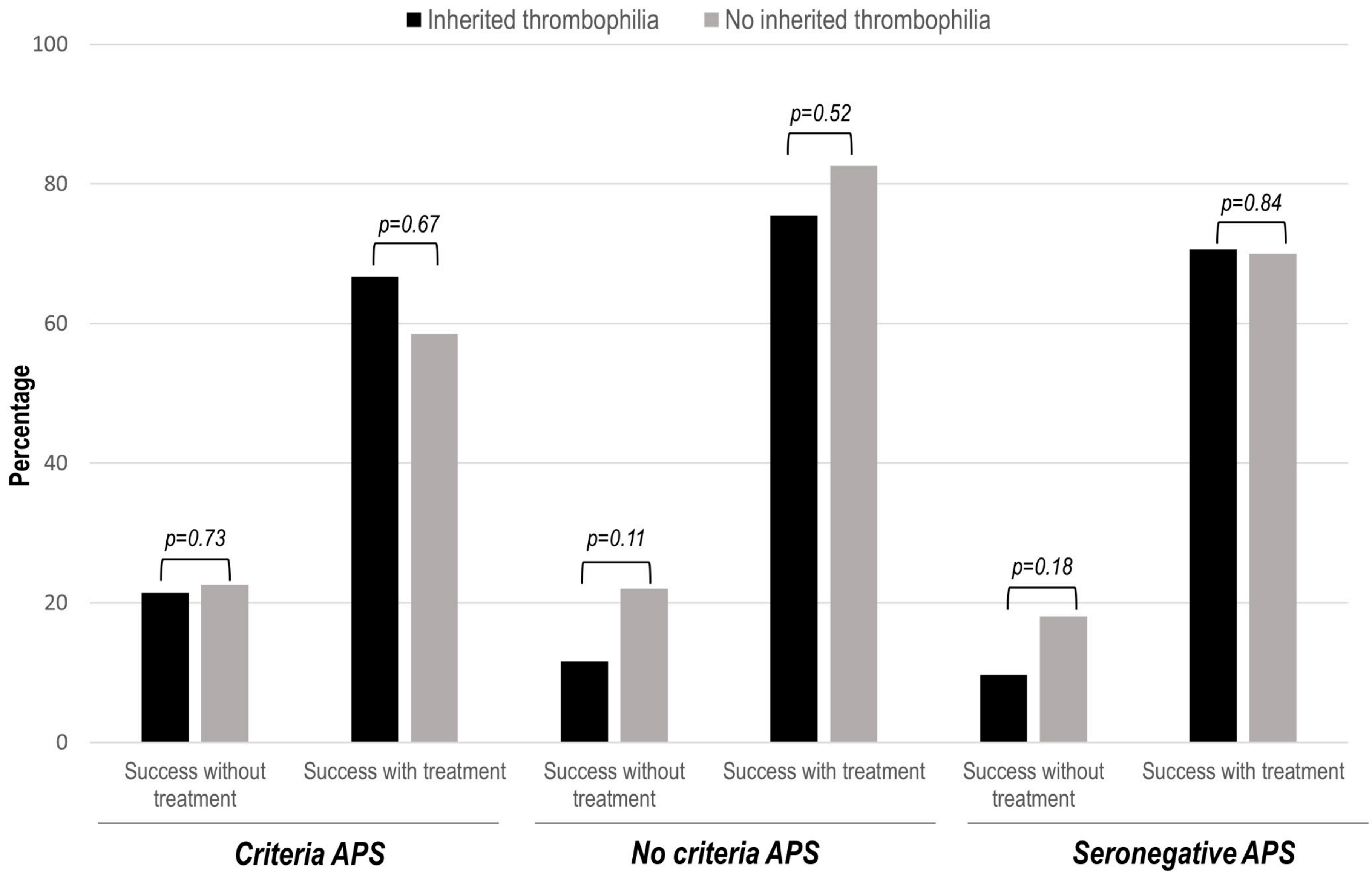

Figure S2. Proportion of successful pregnancy in the study groups after standard treatment according to the different study groups.

Suppl Table S1.- Clinical and serological characteristics in the different study groups.

	**Total N=328**	**Criteria APS N=74**	**Non Criteria APS N=169**	**Seronegative APS N=85**
**APS clinical groups, N (%)**				
**- Fetal death >10 weeks**	60 (18.3)	28 (37.8)	17 (10.1)	15 (17.6)
**- Preterm <34 weeks**	14 (4.3)	9 (12.2)	5 (3.0)	0 (0.0)
**- Abortion <10 weeks (≥3)**	268 (81.7)	50 (67.6)	137 (81.1)	81 (95.3)
**- Thrombosis**	22 (6.7)	14 (18.9)	4 (2.4)	4 (4.7)
**APS serology groups, N (%)**				
**- aCL+ (%)**	48 (14.6)	16 (21.6)	32 (18.9)	0 (0.0)
**- AB2GPI+ (%)**	66 (20.1)	14 (18.9)	52 (30.8)	0 (0.0)
**- LA+ (%)**	49 (14.9)	17 (23.0)	32 (18.9)	0 (0.0)
**- Double/Triple + (%)**	80 (24.4)	27 (36.5)	53 (31.4)	0 (0.0)
**- High risk aPL profile (%)**	124 (37.8)	44 (59.5)	80 (47.3)	0 (0.0)

APS: antiphospholipid síndrome; aCL: anticardiolipin antibodies; AB2GPI: anti beta 2 glycoprotein I; LA: lupus anticoagulant; High-risk aPL profile: Double/Triple+ and/or LA+; aPL: antiphospholipid antibodies.

Supplementary Table S2. Adverse pregnancy outcomes (APO) in patients with and without inherited thrombophilia (IT).

	Total N=328	IT N=45	No IT N=283
APO total, ***N (%)***	310 (94.5)	42 (93.3)	268 (94.7)
Abortion <10 weeks	268 (81.7)	39 (86.7)	229 (80.9)
Fetal death >10 weeks	60 (18.3)	8 (17.8)	52 (18.4)
Preterm <37 weeks	48 (14.6)	2 (4.4)*	46 (16.3)*
Abruptio placentae	7 (2.1)	1 (2.2)	6 (2.1)
Preeclampsia	38 (11.6)	1 (2.2)*	37 (13.1)*

IT: Inherited thrombophilia; APO: adverse pregnancy outcome. *p<0,05.

Supplementary Table S3. Prevalence of inherited thrombophilia (IT) in the general population [7,19–22] and in the group of the present study.

Inherited Thrombophilia	Prevalence General population	Prevalence Study population
Protein S deficiency	0.1-0.2%	6.1%
Protein C deficiency	0.2-0.5%	0.6%
Factor V Leiden	1.6-5%	0.9%
MTFHR	5-15%	3.7%
PT G20210A	1.1-4%	2.4%
Antithrombin deficiency	0.03%	0%
